# Acute presentation of short‑segment Hirschsprung's disease treated with Soave's procedure in a 20‑year‑old male: A case report and mini‑review of the literature

**DOI:** 10.3892/mi.2024.203

**Published:** 2024-11-12

**Authors:** Guillermo Gallardo Chavarría, Raymundo Alfonso Muñoz Cabello, Irene Lizeth Zambrano Loya, Aarón Alberto Ramírez Torres, Ernesto Ramos Martinez

**Affiliations:** 1 Division of Endoscopy and General Surgery, Hospital Angeles Chihuahua, 31217 Chihuahua, Chih., Mexico; 2 Faculty of Medicine and Biomedical Science, Autonomous University of Chihuahua, 31125 Chihuahua, Chih., Mexico; 3 Division of Pathology, Hospital Angeles Chihuahua, 31217 Chihuahua, Chih., Mexico

**Keywords:** colon, Hirschsprung's disease, neural crest cells, adult, Soave's procedure

## Abstract

Hirschsprung's disease is a congenital disorder that affects the motility of the colon. It is caused by the absence of ganglion cells in the large intestinal plexuses. Although uncommon, Hirschsprung's disease can be diagnosed until adulthood. In such cases, this is often due to a history of episodes of chronic constipation since childhood, with the majority of these being misdiagnosed and inadequately treated. The present study describes the case of a 20-year-old male patient, with a history of chronic constipation since childhood managed conservatively. Following acute intestinal obstruction, he required an emergency laparotomy with intestinal resection and colostomy. The histopathological analysis revealed aganglionosis in the sigmoid colon, confirming Hirschsprung's disease. Definitive treatment was subsequently decided with Soave-type surgery, with excellent results obtained in subsequent follow-up sessions. In addition, the present study also provides a mini-review of the literature; several similar reports were identified upon searchingw the literature using the PubMed database. Overall, the present study demonstrates that adults with chronic constipation refractory to treatment should be considered susceptible to the diagnosis of this disease.

## Introduction

Hirschprung's disease is a congenital disease of intestinal motility characterized by the the absence of ganglion cells (aganglionosis) in the myenteric plexus of Auerbach and in the submucosal plexus of Meissner. It extends a variable distance in the colon and arises secondary to a failure in the migration of ganglion cells from the neural crest during embryonic development (1). Although Hirschsprung's disease may have a polygenic influence, the gene most frequently involved is the RET proto-oncogene (2,3). Hirschsprung's disease can be classified according to aganglionic extension into ultra-short segment, short segment, long segment and total colonic aganglionosis (4). Its estimated incidence is 1 in 5,000 newborns, and it is a common cause of intestinal obstruction in newborns and children. Clinical manifestations and diagnosis occur prior to 5 years of age in >90% of cases. However, patients with mild forms of the disease may reach adulthood before the diagnosis is established (5). In adults, the diagnosis of Hirschsprung's disease can only be established after the age of 10 years. There are several case reports describing adults and as it is often missed or misdiagnosed, there is no estimated incidence in adults (4-6). The usual age gap for diagnosis in adults is between 20 and 40 years, with a higher prevalence in males (4,6,7). The present study describes the case of a 20-year-old male patient, with a history of chronic constipation since childhood managed conservatively.

## Case report

**Figure 1:**
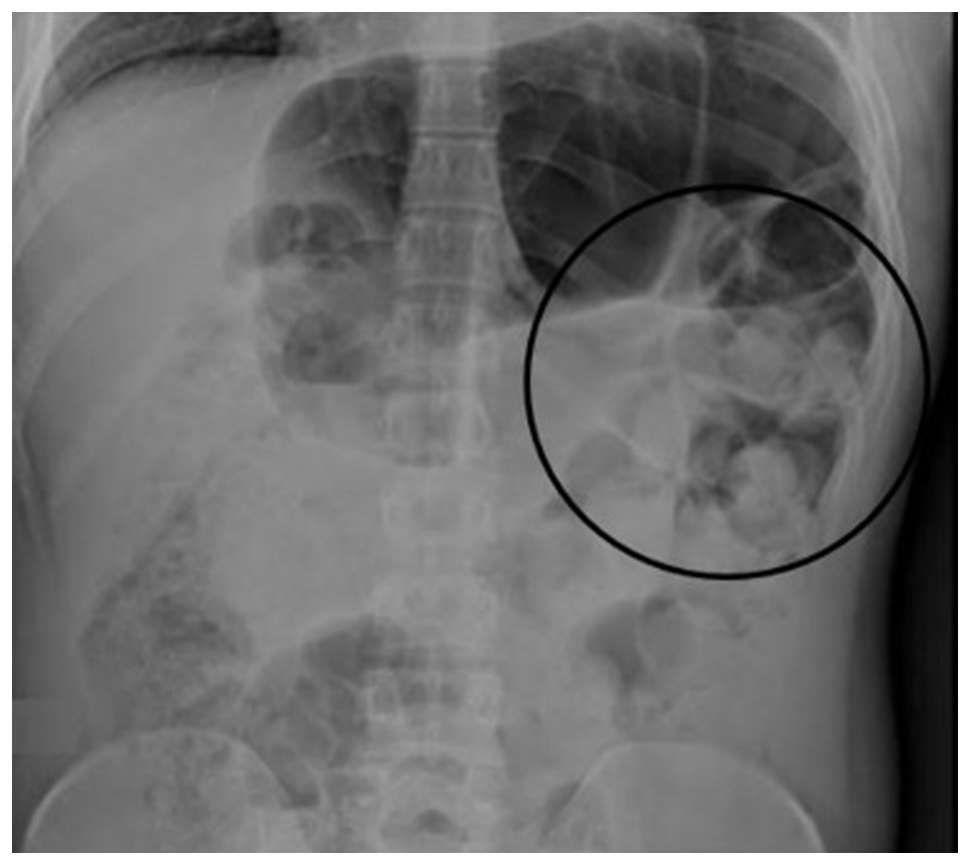
Anteroposterior abdominal X-ray performed with the patient in a standing position. Coprostasis is observed in the entire colonic frame (black circle), with significant gas distension at the level of the splenic angle of the colon.

**Figure 2:**
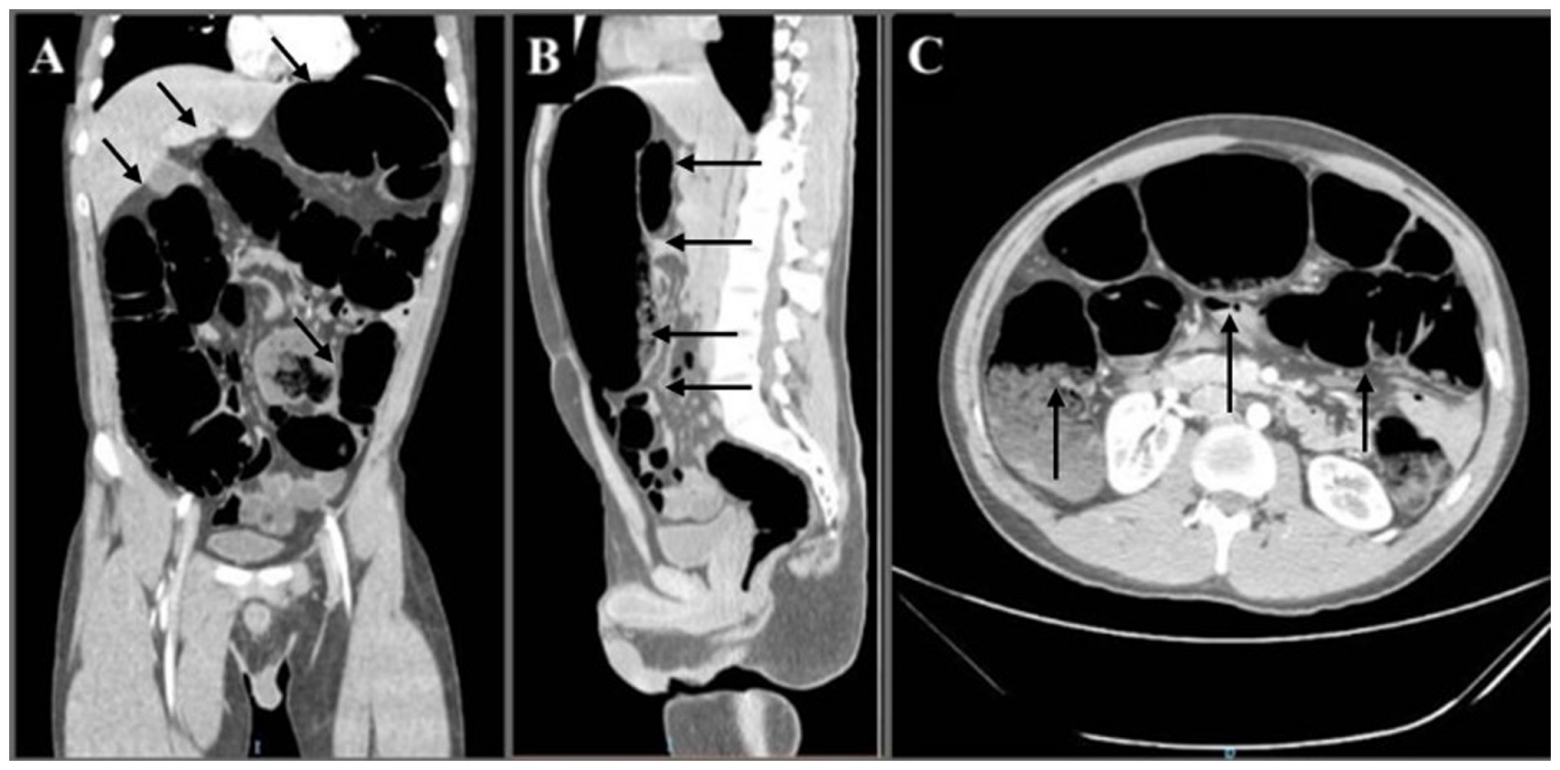
Abdominal computed tomography scan with intravenous contrast. (A) Coronal, (B) sagittal, and (C) axial views. Marked dilatation of the colonic frame with fecal matter residue, with a maximum diameter of up to 9.2 cm and the persistence of gas at the level of the rectum (black narrows) are observed.

A 20-year-old male patient, without a prior medical history, apart from multiple childhood non-relevant and unspecific episodes of constipation, which resolved spontaneously, was admitted to Hospital Angeles Chihuahua (Chihuahua, Mexico). During anamnesis, the patient reported an insidious onset of constipation episodes during his pre-school years. He also reported previous satisfactory resolution by intermittently treatment with *Plantago ovata* (psyllium), laxatives and prokinetics. Therefore, the patient proceeded to self-medicate with the same treatment as necessary, delaying his diagnostic process. Currently, the individual reported absent bowel movements for a duration of 1 week. He also complained that the use of laxatives and enemas had been unsuccessful. At the time of his examination, the symptoms of the patient included anorexia, nausea, biliary emesis and diffuse intractable colicky abdominal pain. Upon a physical examination, a distended abdomen with tenderness to palpation was noted. Therefore, volvulus was suspected and thus, an abdominal X-ray (Fig. 1), and 1 h later an abdominal computed tomography (CT) scan with intravenous contrast were performed (Fig. 2). Following imaging evaluation, emergency surgical intervention was performed due to the risk of bowel perforation.

**Figure 3:**
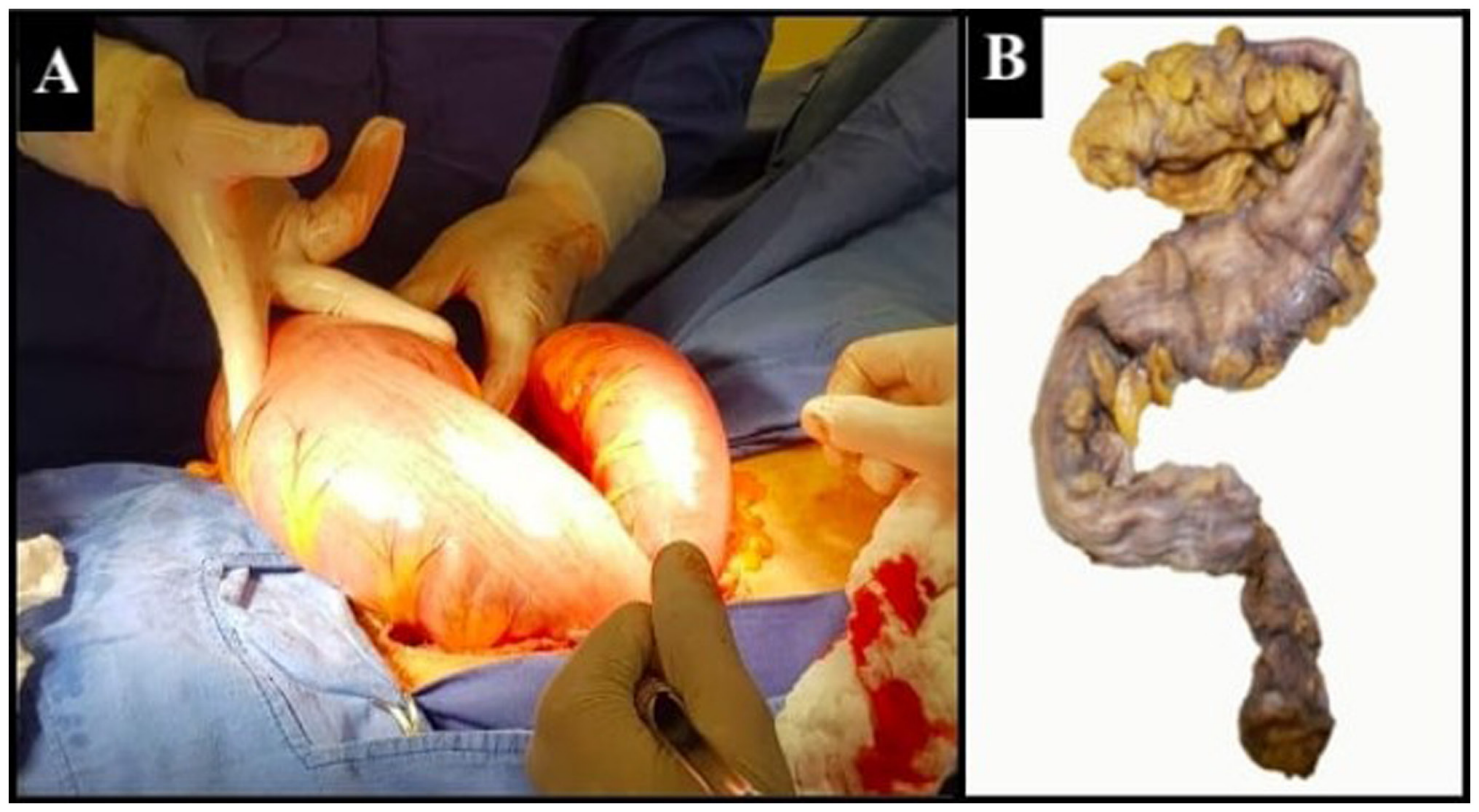
(A) Intraoperative image during the second surgery, where Soave's procedure was performed. (B) Resected sigmoid colon.

**Figure 4:**
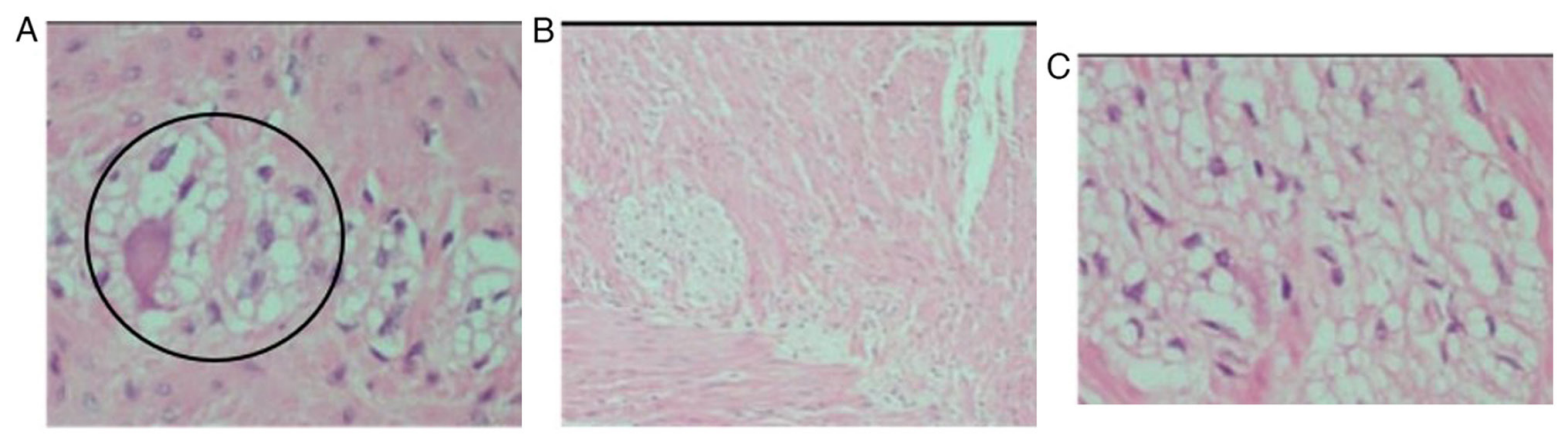
Histopathological analysis, stained with hematoxylin and eosin. (A) Proximal and middle third of the sigmoid colon illustrating submucosal and myenteric plexuses of the muscularis propria with ganglion cells decreased in number or without them (black circles) at x40 magnification. (B and C) Distal 6 cm of the sigmoid colon without ganglion cells in the submucosal and myenteric plexuses at x20 and x40 magnification, respectively.

An exploratory laparotomy was performed, at which time no volvulus was found, and a sigmoid colon distention of 11 cm in diameter was noted (Fig. 3). Seromuscular biopsies were obtained at the descending and sigmoid colon junction, as well as at the sigmoid colon with fragments of the muscular layer. A colostomy was then performed at the descending and sigmoid junction of the colon (8). The samples obtained were processed using 10% buffered formalin, then embedded in paraffin and sectioned into 50-µm-thick slices using a microtome. The sections were placed on slides and stained with hematoxylin and eosin. These procedures were performed at the Cytopathology and Oncological Pathology laboratory, Histopath^®^, in accordance with standard guidelines. The pathology report of the biopsies described the absence of ganglion cells in the myenteric plexus in all the biopsied segments, diagnosing adult Hirschsprung's disease. The patient was subjected to surgery for a second time, at which time Soave's procedure was utilized for colon descent with rectal anastomosis and the resection of the dysfunctional sigmoid colon, and a terminal ileostomy was performed (9). The subsequent histopathological diagnosis confirms the aganglionosis and hypoganglionosis of the sigmoid colon in its last 6 cm, confirming the diagnosis of short-segment adult Hirschprung's disease (Fig. 4). At 2 months following Soave's procedure, a follow-up with a colonoscopy was performed to assess the integrity of the colo-anal anastomosis, where no openings, ulcers, or signs of fistulization were evident. Finally, a third surgery was performed for ileostomy closure (10). Following an uncomplicated post-operative course, the patient was discharged from the hospital. The patient remains uncomplicated at 40 months of follow-up with no difficulty in achieving bowel movements.

## Discussion

Hirschsprung's disease can be classified according to the length of the aganglionic colon from the internal anal sphincter. It is divided into an ultra-short segment when it affects only the internal anal sphincter and <5 cm of the distal rectum, a short segment when the aganglionosis extends to the sigmoid colon (75-80% of cases) (as in the case in the present study), a long segment when the absence of ganglion cells extends beyond the rectosigmoid junction to the splenic flexure or transverse colon (15-20%) and total colonic aganglionosis, affecting the entire colon and less than 50 cm of the ileum (2-13%) (4,11,12).

Of note, >90% of cases are diagnosed in the neonatal period, with suspicion beginning following a delay in meconium elimination for >24 h (13). However, patients with mild forms of the disease may reach adulthood prior to diagnosis, as it is commonly overlooked during medical evaluations and can be masked by effective management of constipation (6,14). The diagnosis of Hirschsprung's disease in adults is relatively difficult compared with that in children, largely due to its infrequency, and the fact that it usually involves short or ultra-short aganglionic segments, which produce mild symptoms that can be managed effortlessly (11). It is estimated that ~2% of cases of chronic constipation in adults could be secondary to Hirschsprung's disease (5). As the causes of chronic constipation can be multifactorial, it is critical to make differential diagnoses and exclude other causes of chronic constipation and megacolon in adults, such as: Colorectal cancer, volvulus, iatrogenic causes, enteric neuropathies, stenosis, colonic motility slowing, Chagas disease, anatomical or functional (endocrine or metabolic disorders) obstruction to defecation or idiopathic megacolon (11,15).

In addition, since the most important etiopathogenetic factor in the development of Hirschsprung's disease is a neuronal disorder, this pathology can be classified as an intestinal neuropathy (16). The clinical presentation of intestinal neuropathies varies significantly according to the degree of biochemical disruption, neuronal connectivity and cell density present in the enteric ganglia (17). Sometimes, in Hirschsprung's disease, the distinction between innervated and aganglionic segments is not well-defined. As a result, transitional zones can exist where the cellular density and characteristics of the ganglia exhibit considerable variability. These incomplete enteric ganglia contribute to the formation of hybrid structures, leading to greater heterogeneity in the clinical manifestations of Hirschsprung's disease. This variability in ganglion cell density may further complicate the histopathological diagnosis of the condition (18). Indeed, apart from cases of complete aganglionosis in Hirschsprung's disease, the degree of neuronal dysfunction or the phenotype of the pathology directly depends on the genetic and epigenetic interactions of the patient. In a number of instances, there are groups of immature or inactive neurons in the colonic plexuses, which may either activate or deactivate, thereby exacerbating or alleviating the symptoms of the disease depending on various environmental factors (19).

As there is a wide array of differential diagnoses in adults, and the onset of symptoms can also occur at a late stage and be minimal, a high index of suspicion and a detailed history of constipation characteristics since childhood are required. The most common initial presentation of Hirschsprung's disease in adults consists of insidious and intermittent episodes of constipation of variable duration, bloating and abdominal pain (4,6,7). Adults mostly seek medical consultation for chronic refractory constipation from childhood onwards, complaints of abdominal pain and distension, and regular use of laxatives or enemas. This disease is frequently misdiagnosed in adults as chronic constipation without conclusive analyses, as constipation is the most common symptom in this population with a prevalence of 10 to 15%. Late diagnoses are particularly associated with short segments (<10 cm in length) or when symptoms are attenuated by the chronic use of laxatives, a low-residue diet, antispasmodics and other treatments (13). Evidence of this has been previously reported in the literature. There is a documented case of a 9-year-old patient with Hirschsprung's disease, who was diagnosed only at that age due to having bowel movements approximately every 3 to 4 days. At the same time, the patient exhibited a favorable response to the use of laxatives and prokinetics (20). Similarly, a 19-year-old Caucasian woman with a chronic history of constipation, managed daily with enemas and laxatives, was diagnosed with Hirschsprung's disease at a later stage. The diagnosis was confirmed through a biopsy of the colonic tissue, which revealed residual neuronal tissue (5).

However, as in the case described herein, the presentation of Hirschsprung's disease in some adults can be acute, requiring laparotomy and rapid surgical management due to overlying intestinal occlusive syndrome (4,7,6,21). Although there are well-established tests for the diagnosis of Hirschsprung's disease, due to the rarity of its occurrence in adults, these are rarely performed. Nevertheless, when Hirschsprung's disease is suspected in an adult, the same tests performed in pediatric patients can also be performed in adults. Some of these tests include plain abdominal radiographs, contrast enema, anal manometry and full-thickness rectal biopsies (5,9,22). An initial assessment can be performed with simple abdominal radiography, where a grossly distended colon, the abnormal distribution of intestinal gas and the absence of stool in the rectum could be observed (9,23).

The most effective imaging method to assess Hirschsprung's disease is contrasted enema (sensitivity, 70%; specificity, 83%), in which an observable transition sign (the narrow zone proximal to the anus, funnel-shaped transition zone and dilated zone correspond to the aganglionic, hypoganglionosis and normal ganglionic zones, respectively) is the most important criterion to help confirm a suspicion for Hirschsprung's disease (24). Anorectal manometry is another study modality with high sensitivity (91%) and specificity (93%) that can be used to evaluate suspected cases. It consists of rectal distension using a pressure-controlled balloon with the subsequent measurement of contraction/relaxation of the interior and exterior anal sphincters. An abnormal test consistent with Hirschsprung's disease is a failure of the internal anal sphincter to relax after rectal distension (23,25). However, despite all the tests available, the gold standard for a definitive diagnosis is a histopathological analysis. The diagnosis of Hirschsprung's diseases is made when the absence of ganglion cells and submucosal nerve hypertrophy are found (4,5,23,25).

Definitive treatment is surgical and is focused on elimination of the aganglionic segment of the colon with subsequent anastomosis distal to the affected area (1,22). There are different surgical techniques to treat Hirschsprung's disease in adults; however, when selecting one, the decision should be individualized based on the clinical status of the patient and the classification according to the length of the affected segment (26). Fecal diversion procedures (e.g., colostomy) tend to be applied to older patients and with an American Society of Anesthesiologists (ASA) score of ≥3, which probably explains a higher incidence of post-operative complications, pre-operative gastrointestinal organic lesion and the duration of obstruction. On the other hand, patients who underwent an intestinal continuity restoration technique were more likely to be younger and with a better ASA score of 1-2, which translated into a shorter length of hospitalization and fewer complications (26,27).

There are three main surgical techniques described: Swenson (rectosigmoidectomy), Duhamel (retrorectal transanal pull-through), Lynn and Soave (endorectal pull-through) (1,7). The Soave technique was first described in 1960 and consists of a colo-anal anastomosis above the dentate line with rectal mucosectomy. Its technical advantage over the other techniques consists of the greater preservation of the internal sphincter, vessels and pelvic nerves (4,7). Currently, both Soave's original procedure and the modified one by Jarry and Faucheron (28) can be performed minimally invasively via laparoscopy, with excellent long-term results.

**Figure 5:**
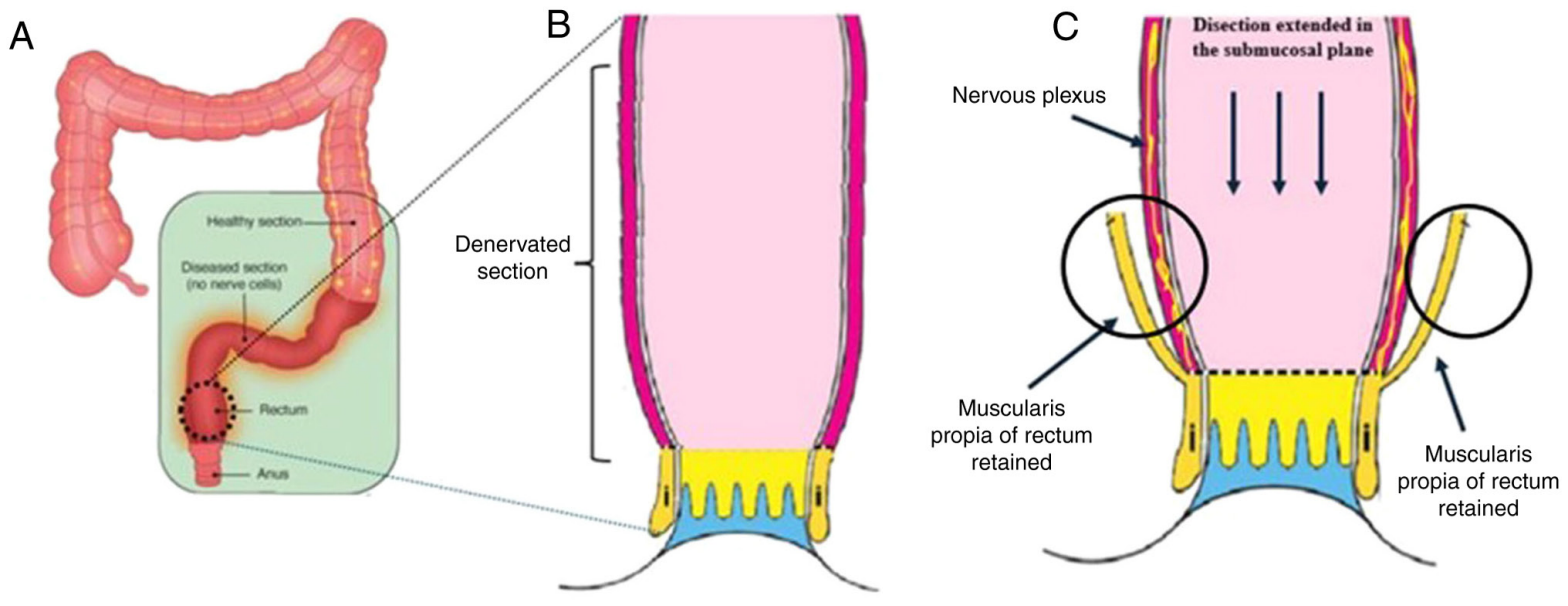
Illustrative diagram of the pathophysiology of Hirschsprung's disease and its treatment. (A) Large intestine in a person affected by Hirschsprung's disease. The loss of innervation in the nervous plexuses, characteristic of the disease, is observed in the terminal region of the descending colon and rectum. (B) Magnified illustration of the terminal region of the descending colon and rectum in a person with Hirschsprung's disease. The absence of innervation in the submucosal layers of the tissue is clear. (C) Schematic representation of Soave surgery to alleviate the symptoms in Hirschsprung's disease. In Soave procedure, the initial radial incision is made through the mucosa, and the dissection is extended proximally in the submucosal plane for several centimeters before dividing the muscularis propria. Therefore, the muscularis propria of the distal rectum is retained as a ‘cuff’ (black circles).

Regardless of the surgical treatment employed, patients with Hirschsprung's disease who undergo colonic resection may develop chronic colitis as a chronic adverse effect, rendering the choice of treatment complex (29). Factors, such as continence and the maintenance of intestinal physiology are key parameters for evaluating therapeutic efficacy. The Soave, Duhamel and transanal endorectal pull-through procedures do not exhibit differences concerning the post-operative nutritional status of patients with Hirschsprung's disease (30). On the other hand, the Soave procedure is preferred for preserving the integrity of pelvic structures to a greater extent, although it has also been shown to be associated with an increased incidence of enterocolitis (31). Furthermore, the Soave procedure is superior to the Duhamel procedure in preventing both fecal incontinence and symptoms related to the post-operative period (32). As regards intestinal physiology, the Soave procedure does not exhibit differences compared to other treatments (33). Therefore, the Soave technique appears to be an ideal treatment within the context of limited resources for managing adult Hirschsprung's disease, as is the case in the present study (Fig. 5) (14,34).

According to the European Reference Network for rare Inherited and Congenital Anomalies (ERNICA) guidelines (29), a recommended pre-operative preparation for elective surgery includes a combination of saline rectal irrigations one to three times a day (effective in 75% of cases) and a single dose of antibiotics to reduce the risk of wound infections (27). Even though surgical treatment remains the first and most effective treatment option, and techniques have greatly improved over time, there are still a number of post-operative complications that could greatly decrease quality of life of patients. Possible therapies focusing on genetic engineering, stem cell research and tissue engineering could be readily available in the future (3).

Finally, concerning long-term outcomes, the patient described in the present study demonstrated favorable fecal continence, with a bowel pattern of approximately one evacuation daily or every other day, as reported by the patient. However, the authors consider that a thorough evaluation of the effects of surgery on intestinal physiology over an extended period of time is necessary to accurately assess the long-term efficacy of the intervention.

In conclusion, Hirschsprung's disease is a rare cause of chronic constipation in adults. In the majority of cases, it causes subtle and sporadic episodes of constipation, bloating and abdominal pain; however, an acute presentation of intestinal obstruction that requires urgent surgical management can be observed in a minority of patients. As differential diagnoses are very varied and may be multifactorial, a high index of suspicion is required. Special emphasis should be placed on past episodes of constipation, including those in childhood. Hirschsprung's disease may be the underlying cause of an acute episode of intestinal occlusive syndrome; hence, taking multiple intraoperative biopsies of the colorectum for further analyses in those who undergo urgent laparotomy without an apparent cause identified appears to be a feasible option. This should be specially considered in patients with prior episodes of constipation, since an early age without a definite diagnosis established. The patient required urgent laparotomy and colostomy due to overlying intestinal occlusive syndrome with risk of bowel perforation. Multiple seromuscular biopsy samples were obtained, as there was no evident cause of intestinal obstruction identified, aiding in the diagnosis of Hirschsprung's disease. Definitive resolution with excellent long-term results was later achieved using Soave's procedure.

## Data Availability

The datasets used and/or analyzed during the current study are available from the corresponding author on reasonable request.
